# Nuclear PRMT5, cyclin D1 and IL-6 are associated with poor outcome in oropharyngeal squamous cell carcinoma patients and is inversely associated with p16-status

**DOI:** 10.18632/oncotarget.14682

**Published:** 2017-01-17

**Authors:** Bhavna Kumar, Arti Yadav, Nicole V. Brown, Songzhu Zhao, Michael J. Cipolla, Paul E. Wakely, Alessandra C. Schmitt, Robert A. Baiocchi, Theodoros N. Teknos, Matthew Old, Pawan Kumar

**Affiliations:** ^1^ Department of Otolaryngology-Head and Neck Surgery, The Ohio State University, Columbus, OH 43210 USA; ^2^ The Ohio State University Comprehensive Cancer Center, Columbus, OH 43210 USA; ^3^ Center for Biostatistics, The Ohio State University, Columbus, OH 43210 USA; ^4^ Department of Pathology, The Ohio State University, Columbus, OH 43210 USA; ^5^ Department of Internal Medicine, The Ohio State University, Columbus, OH 43210 USA; ^6^ Department of Pathology and Laboratory Medicine, Emory University School of Medicine, Atlanta, GA 30303 USA

**Keywords:** PRMT5, OPSCC, HPV, cyclin D1, IL-6

## Abstract

Protein arginine methyltransferase-5 (PRMT5) plays an important role in cancer progression by repressing the expression of key tumor suppressor genes via the methylation of transcriptional factors and chromatin-associated proteins. However, very little is known about the expression and biological role of PRMT5 in head and neck cancer. In this study, we examined expression profile of PRMT5 at subcellular levels in oropharyngeal squamous cell carcinoma (OPSCC) and assessed its correlation with disease progression and patient outcome. Our results show that nuclear PRMT5 was associated with poor overall survival (*p* < 0.012) and these patients had 1.732 times higher hazard of death (95% CI: 1.127–2.661) as compared to patients in whom PRMT5 was not present in the nucleus of the tumors. Nuclear PRMT5 expression was inversely correlated with p16-status (*p* < 0.001) and was significantly higher in tumor samples from patients who smoked > 10 pack-years (*p* = 0.013). In addition, nuclear PRMT5 was directly correlated with cyclin D1 (*p* = 0.0101) and IL-6 expression (*p* < 0.001). In a subgroup survival analysis, nuclear PRMT5-positive/IL-6-positive group had worst survival, whereas nuclear PRMT5-negative/IL-6-negative group had the best survival. Similarly, patients with p16-negative/nuclear PRMT5-positive tumors had worse survival compared to patients with p16-positive/nuclear PRMT5-negative tumors. Our mechanistic results suggest that IL-6 promotes nuclear translocation of PRMT5. Taken together, our results demonstrate for the first time that nuclear PRMT5 expression is associated with poor clinical outcome in OPSCC patients and IL-6 plays a role in the nuclear translocation of PRMT5.

## INTRODUCTION

Head and neck squamous cell carcinoma (HNSCC) is the eighth most frequent cancer worldwide and five-year survival rate (< 50%) is among the lowest of the major cancers [1, 2]. Tobacco usage and alcohol consumption have been known to be the strongest risk factors for the development of this disease [3]. However, it is now being recognized that human papillomavirus (HPV) can also play a role in the development of a subset of head and neck cancers [4]. Most of the HNSCC are diagnosed in advanced stages and the outcome of these patients is often poor [5]. Five year survival rates for patients with early stage localized head and neck cancers are more than 80%, but drop to 40% when the disease has spread to the neck nodes, and to below 20% for patients with distant metastatic disease [5–7]. Patients with head and neck cancers encompass a heterogeneous group and can be further subdivided into two distinct tumor subtypes; human papillomavirus (HPV)-negative and HPV-positive tumors [8]. HPV-positive tumors predominantly arise in the oropharynx and majority of these patients with HPV-positive tumors respond very well to traditional chemo-radiotherapy and demonstrate significantly favorable clinical outcomes [9, 10]. However, there is a small subset of HPV-positive patients that do not respond well to standard therapy and show markedly poor clinical outcome [11, 12]. In contrast to HPV-positive patients, the majority of HPV-negative patients are usually smokers, have more aggressive disease and many of these patients develop resistance to chemotherapy leading to poor prognosis [10]. Therefore, it is imperative that novel therapeutic targets are identified to increase the long-term survival of these patients as well as decrease the adverse effects associated with standard chemo-radiation regimens [13].

Recent studies have shown that protein arginine methyltransferases (PRMTs) regulate a number of cellular processes, including proliferation, apoptosis, anoikis and epithelial-mesenchymal transition (EMT), which play an important role in tumor growth and metastasis [14–18]. There are nine members in the PRMT family in humans which are subdivided into 3 types based on their distinct methylation characteristics [19]. PRMT2, 3, 6, 7 and 8 exhibit tissue specific expression, whereas PRMT1, 4 and 5 are more universally expressed [20]. PRMT5 is the major type II enzyme that mediates post-translational arginine symmetric dimethylation and it was originally identified as a transcriptional repressor [19, 21]. PRMT5 methylates a host of transcriptional factors (E2F1, p53, FEN1, HOXA9, RAD9 etc.) and chromatin-associated proteins (H2AR3, H3R2, H3R8, H4R3 etc.) to mediate its oncogenic function by repressing the expression of regulatory suppressor genes [18, 22–26]. The transcriptional repressor function of PRMT5 is also critical for the tumor cell release from the primary tumor through the epithelial-mesenchymal transition (EMT) and tumor metastasis. A hallmark of EMT is the loss of E-cadherin expression, which in turn is regulated by the transcription factor Snail. Recently, PRMT5 was shown to repress E-cadherin expression by interacting with Snail through a bridging molecule Ajuba [17]. PRMT5 also regulates cell cycle progression by increasing the expression of positive regulators of G1 phase (Cyclin D1, cyclin D2 and CDK4) and decreasing the expression of negative regulators of G1 (Rb)[27]. Recent studies have shown that PRMT5 is overexpressed in a number of cancer types including ovarian, lung, colon, gastric and bladder cancer and is associated with poor clinical outcome [28–30]. However, there is no reported study that has examined PRMT5 expression, particularly subcellular localization, in a large cohort of tumors from head and neck cancer patient population and its correlation with patient outcome.

In this study, we examined the expression and localization of PRMT5 in oropharyngeal squamous cell carcinoma (OPSCC) tumors and correlated it with patient survival, p16 status (an established surrogate for tumor HPV status in OPSCC), smoking status, cyclin D1 expression, IL-6 expression and other clinical and pathological variables. Our results show a direct correlation between nuclear PRMT5 expression and poor overall survival. Nuclear PRMT5 expression was significantly higher in tumor samples from patients that smoked > 10 pack-years and inversely correlated with p16 status. In addition, nuclear PRMT5 expression directly correlated with IL-6 and cyclin D1 expression. In our mechanistic experiments, IL-6 treatment markedly enhanced nuclear translocation of PRMT5 in head and neck cancer cells.

## RESULTS

**Figure 1:**
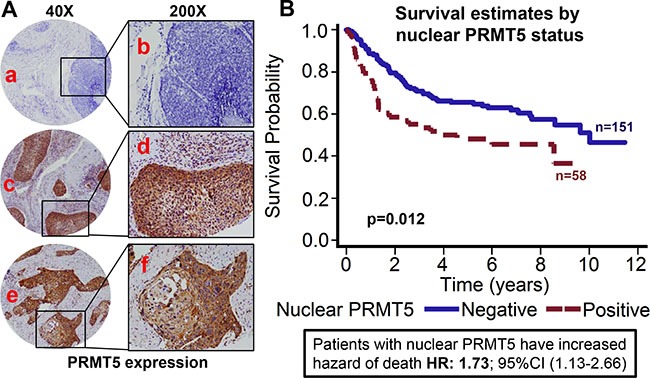
Nuclear PRMT5 expression is associated with poor overall survival in OPSCC Tissue microarrays (TMAs) containing primary tumor samples from OPSCC patients were stained for PRMT5 expression. (**A**) Representative pictures of tumor cores negative for PRMT5 expression (a and b), positive for nuclear PRMT5 expression (c and d), and negative for nuclear PRMT5 expression (positive for cytosolic PRMT5 expression, e and f). (**B**) Overall survival estimates of patients according to nuclear PRMT5 expression.

To evaluate the expression pattern and clinical importance of PRMT5, cyclin D1 and IL-6 in OPSCC, we assessed the expression of these biomarkers in TMA's constructed using 211 surgically treated samples. Patient characteristics are listed in Table 1. Representative images of staining for PRMT5 and cyclin D1 are included in Figures 1 and 4, respectively. p16 was used as surrogate marker for HPV status. 158 tumors (75.2%) were p16 positive and 52 tumors (24.8%) were negative. The median follow-up time was 5.5 years (range 0.1–11.5). The 5-year survival rates for the whole group were 60.6%; 69.6% for the p16-positive group and 32.6% for the p16-negative group.

**Table 1:** Patient Characteristics and Demographics

Patient Characteristics	*n*	%
**Age (years), mean (SD)**	57.7	9.7
**Marital Status**		
Single/Divorced/Widowed	87	45.6
Married	104	54.5
**Race**		
African American/Black	9	4.3
White	202	95.7
**Sex**		
Female	45	21.3
Male	166	78.7
**Smoking Status**		
≤ 10 pack years	51	25.1
> 10 pack years	152	74.9
**Extracapsular Spread**		
No	118	57.8
Yes	86	42.2
**p16 Status**		
Negative	52	24.8
Positive	158	75.2
**Mucosal Margins**		
Free of Carcinoma	174	83.7
Positive Margins	34	16.4
**Primary site**		
BOT	52	24.8
Tonsil	141	67.1
Other	17	8.1
**Node Stage**		
N0	28	13.3
N1	48	22.8
N2	126	59.7
N3	9	4.3
**Perineural Invasion**		
No	157	74.8
Yes	53	25.2
**AJCC Stage**		
I	5	2.4
II	11	5.2
IIII	51	24.2
IV	144	68.3
**Tumor Stage**		
T1	45	21.3
T2	86	40.8
T3	41	19.4
T4	39	18.5

### Nuclear PRMT5 expression is directly associated with poor overall survival and inversely associated with p16 status

**Figure 2:**
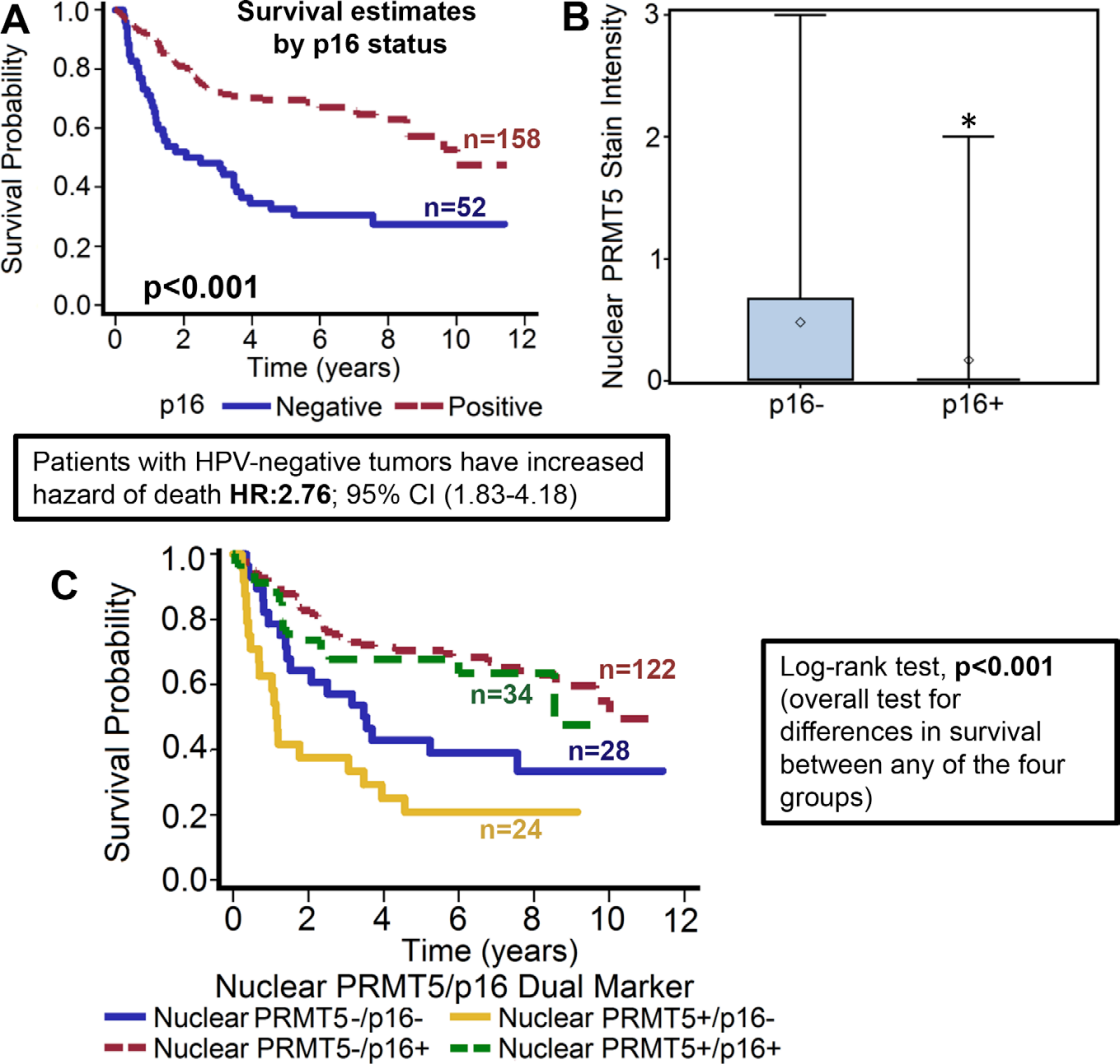
Nuclear PRMT5 expression is significantly lower in p16-positive tumors as compared to p16-negative tumors (**A**) Overall survival estimates of patients according to p16 status. (**B**) Nuclear PRMT5 expression in p16-negative versus p16-positive tumors. *represent a significant difference in nuclear PRMT5 expression in p16-positive (p16+) tumor samples as compared to p16-negative (p16-) tumor samples. (**C**) Survival estimates of patients according to nuclear PRMT5 expression and p16 status.

PRMT5 expression was evaluable in 209 tumors and it was predominately cytoplasmic in the OPSCC tumor samples (72.2%; 151/209). However, we also observed nuclear PRMT5 expression in 58/209 (27.8%) tumors. Tumors that expressed PRMT5 in the nucleus were categorized as nuclear positive and tumors with cytoplasmic expression were classified as nuclear negative. Higher nuclear PRMT5 expression was associated with poor overall survival (*p* < 0.012). Patients whose tumors were nuclear PRMT5 positive had a 1.732 hazard ratio of death (95% CI: 1.127–2.661) as compared to those in which PRMT5 was not present in the nucleus (Figure 1A–1B). The poorer prognosis of patients with tumors expressing nuclear PRMT5 was maintained after adjustments for age, T stage, N stage, AJCC stage, gender and smoking status (*p* = 0.0055; HR 1.912 95% CI: 1.210–3.022). We and others have previously shown that OPSCC patients with HPV-negative tumors have inferior outcomes as compared to patients with HPV-positive patients [9, 10]. In this present study, we also observed that patients with p16-negative tumors have significantly higher hazard of death (HR: 2.76; 95% CI 1.826–4.184) as compared to patients with p16-positive tumors (Figure 2A). This poor prognosis of patients with p16-negative tumors was maintained after adjustments for age, T stage, N stage, AJCC stage, gender and smoking status (*p* = 0.0043; HR 2.073 95% CI:1.257–3.418). Nuclear PRMT5 expression was inversely associated with p16 expression. A greater proportion of p16-negative tumors expressed higher nuclear PRMT5 as compared to p16-positive tumors (58.65% versus 16.35, Figure 2B, *p <* 0.001). In the subgroup survival analysis, p16-negative/PRMT5 nuclear-positive (p16-/Nuclear PRMT5+) group had the worst survival compared to the other groups (Figure 2C, *p* < 0.001).

**Figure 3:**
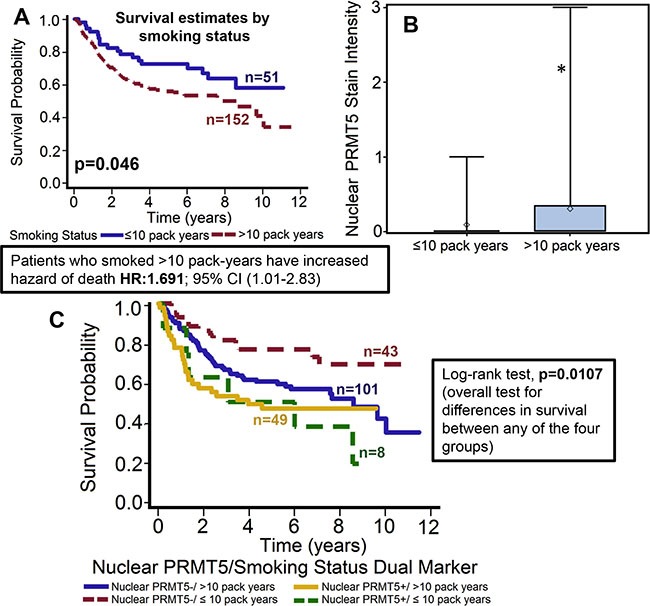
Patients who smoked > 10 pack-years had significantly poor overall survival and markedly higher nuclear PRMT5 expression (**A**) Overall survival estimates of patients according to smoking status. (**B**) Nuclear PRMT5 expression in tumor samples from patients that smoked ≤ 10 pack-years or > 10 pack-years. *represent a significant difference in nuclear PRMT5 expression in patients that smoked < 10 pack-years as compared patients that smoked > 10 pack-years. (**C**) Overall survival estimates according to nuclear PRMT5 expression and smoking status.

Patients who smoked > 10 pack-year showed poor overall survival and had significantly higher expression of nuclear PRMT5. We and others have previously shown that smokers have poor clinical outcome as compared to non-smokers [10, 31]. In this study, our results further corroborate those finding and show that patients who smoked > 10 pack-year had significantly poor overall survival as compared to patients who smoked ≤ 10 pack-year (*p* = 0.046, Figure 3A). The hazard of death for patients who smoked > 10 pack-years was 1.691 (95% CI: 1.009–2.832). In addition, smokers (> 10 pack-years) had significantly higher nuclear PRMT5 expression (Figure 3B, *p* = 0.013). A larger proportion of patients who smoked > 10 pack-years were positive for nuclear PRMT5 expression as compared to patients that smoked ≤ 10 pack-years (24.38% versus 3.98%). In the subgroup survival analysis, PRMT5 nuclear-negative/≤ 10 pack years group had the best survival compared to the other groups (Figure 3C, *p* = 0.0107).

### Cyclin D1 expression is associated with poor overall survival and is directly correlated with nuclear PRMT5 expression

**Figure 4:**
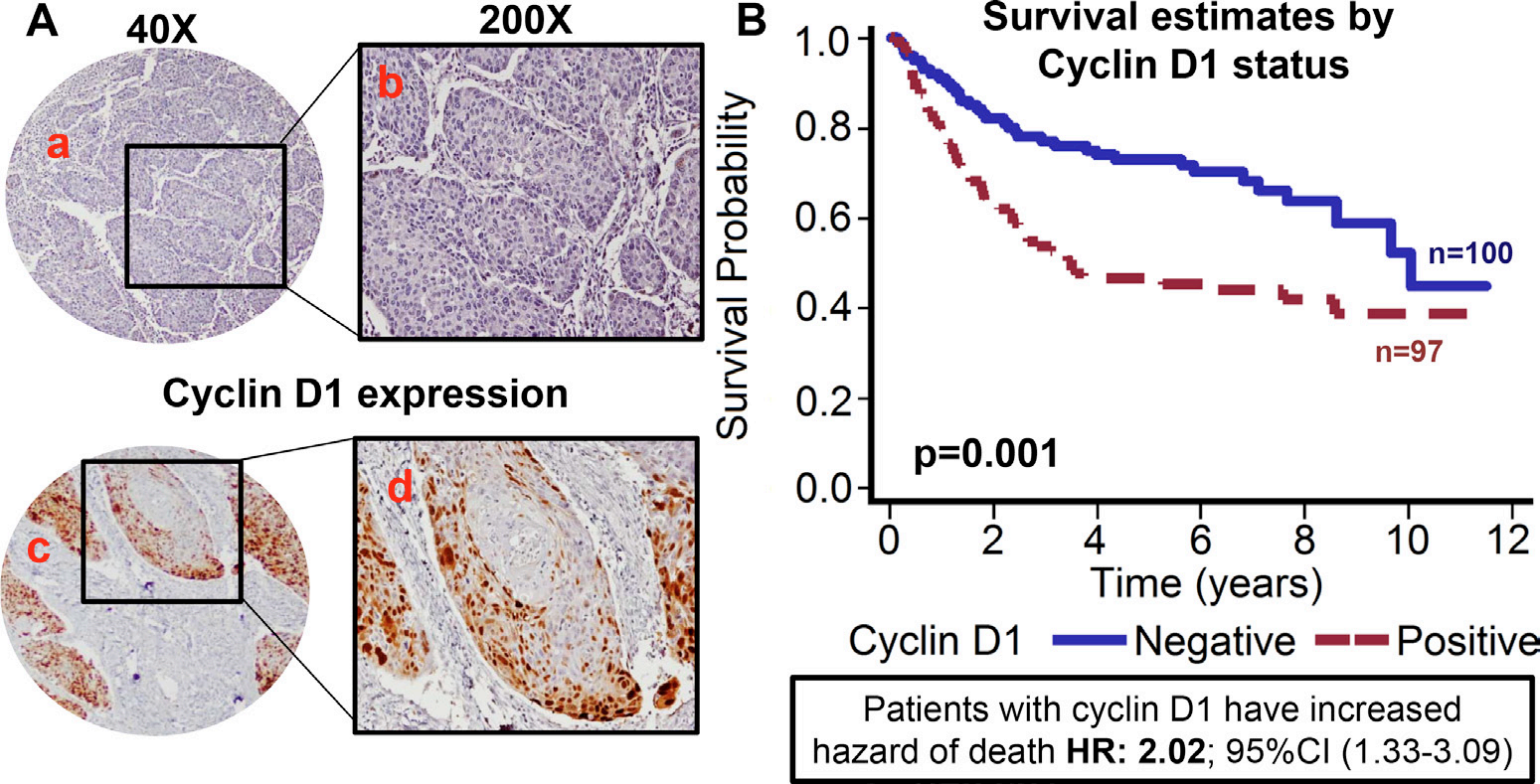
Cyclin D1 expression is associated with poor overall survival TMAs were stained for cyclin D1 expression. (**A**) Representative pictures of tumor cores negative for cyclin D1 expression (a and b) and positive for cyclin D1 expression (c and d). (**B**) Overall survival estimates of patients according to cyclin D1 expression.

**Figure 5:**
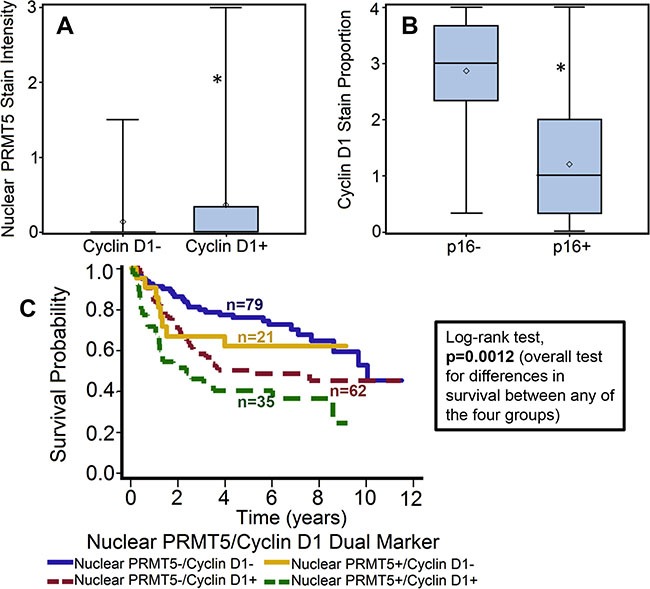
Cyclin D1 expression is directly correlated with nuclear PRMT5 expression and inversely with p16 status (**A**) Nuclear PRMT5 expression in tumor samples with cyclin D1-negative (cyclin D1-) or cyclin D1-positive (cyclin D1+) expression. *represent a significant difference in nuclear PRMT5 expression in tumor samples with cyclin D1-positive or cyclin D1-negative expression. (**B**) Cyclin D1 expression in p16-negative versus p16-positive tumors. *represent a significant difference in cyclin D1 expression in tumor samples with p-positive as compared to p-negative tumors. (**C**) Overall survival estimates according to nuclear PRMT5 and cyclin expression.

Recent studies have shown that PRMT5 regulates tumor growth by modulating cyclin D1 expression [27]. In addition, amplification of cyclin D1 gene and overexpression of cyclin D1 protein has been reported in HNSCC [32]. Cyclin D1 expression was evaluable in 197 OPSCC tumors. Patients with tumor that overexpressed cyclin D1 showed poor overall survival as compared to patients with tumor that were negative for cyclin D1 expression (Figure 4A–4B). The hazard of death for patients with positive cyclin D1 expression was 2.025 (95% CI: 1.326–3.092). This poorer prognosis of patients with tumors expressing higher cyclin D1 was maintained after adjustments for age, T stage, N stage, AJCC stage, gender and smoking status (*p* = 0.0009; HR 2.209 95% CI: 1.385–3.523). Patients whose tumors were nuclear PRMT5 positive had higher expression of cyclin D1 (*p* = 0.01, Figure 5A). Similarly, patients whose tumors were p16-negative had higher expression of cyclin D1 (*p* < .001, Figure 5B). In the subgroup survival analysis, PRMT5 nuclear-positive and cyclin D1-positive (nuclear PRMT5+/cyclin D1+) group had the worst survival compared to the other groups (Figure 5C). In addition, cyclin D1 expression was directly associated with tumor recurrence (*p* = 0.04).

### IL-6 overexpression is associated with poor overall survival and is directly correlated with nuclear PRMT5 expression

**Figure 6:**
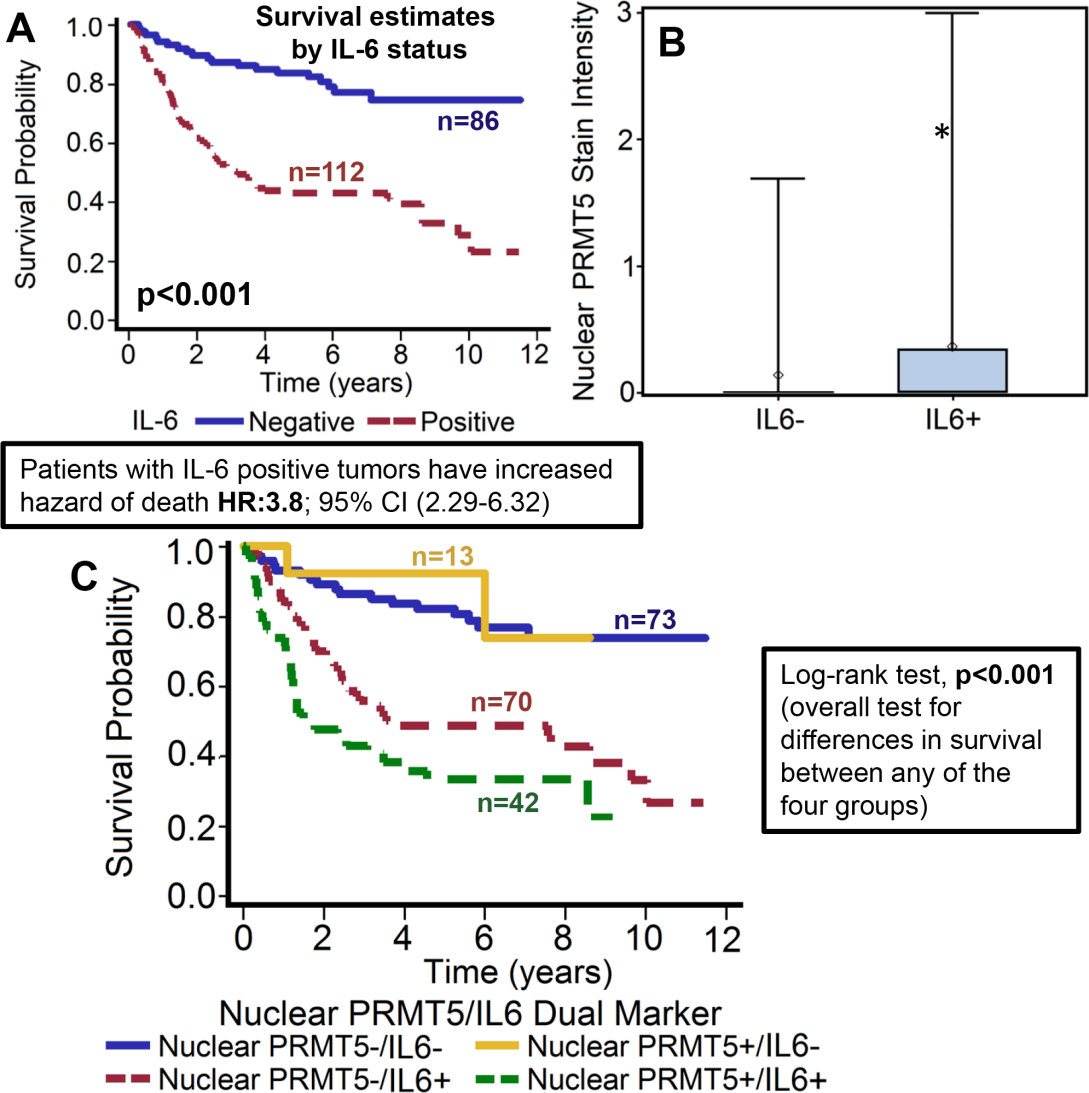
High IL-6 expression is associated with poor overall survival and directly correlates with nuclear PRMT5 expression (**A**) Overall survival estimates of patients according to IL-6 expression. (**B**) Nuclear PRMT5 expression in IL-6-negative (IL-6-) or IL-6-positive (IL-6+) tumor samples. (**C**) Overall survival estimates according to nuclear PRMT5 expression and IL-6 expression.

IL-6 expression was evaluable in 198 tumors and was found to be overexpressed in 112 (56.6%) tumors. Overexpression of IL-6 was significantly associated with worse overall survival of patients with OPSCC (*p* < 0.001) [33]. The hazard of death for patients with positive IL-6 expression was 3.8 (95% CI: 2.287–6.324, Figure 6A). This poorer prognosis of patients with tumors expressing higher IL-6 was maintained after adjustments for age, T stage, N stage, AJCC stage, gender and smoking status (*p* < 0.0001; HR 4.089 95% CI: 2.390–6.995). Interestingly, nuclear PRMT5 expression was directly associated with high IL-6 expression in the primary tumor samples (*p* < 0.001; Figure 6B). In the subgroup survival analysis, IL-6+/nuclear PRMT5+ group had the worst survival as compared to IL-6-/nuclearPRMT5+ and IL-6-/nuclear PRMT5- groups (*p* < 0.001, Figure 6C). In addition, IL-6 overexpression was also directly associated with tumor recurrence (*p* < 0.001), perineural invasion (*p* = 0.007), extracapsular spread (*p* = 0.014) and inversely associated with p16 status (*p* = 0.011).

### IL-6 promotes nuclear translocation of PRMT5

**Figure 7:**
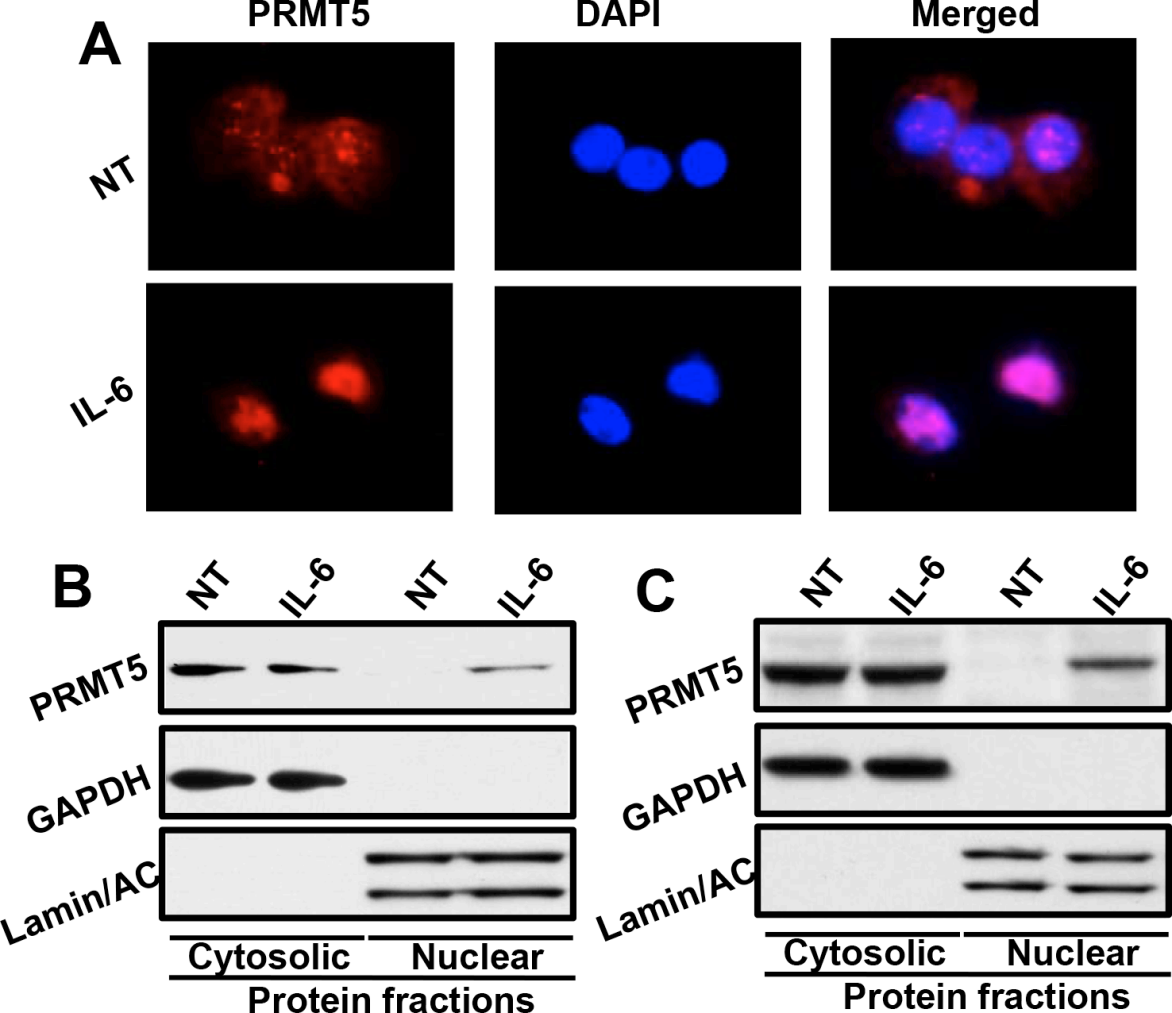
IL-6 promotes PRMT5 nuclear translocation (**A**) CAL27 cells were cultured in Labtech chambers and treated with IL-6 for 30 minutes. Cells were then stained for PRMT5 (red) and nucleus (DAPI, blue) and photographed at 600X. (**B**–**C**) CAL27 (B) or UM-SCC-74B (C) cells were treated with IL-6 for 30 minutes. Nuclear and cytosolic fractions were isolated from these cells and Western blotted for GAPDH, Lamin A/C and PRMT5.

Our results from this study show that nuclear localization of PRMT5 was directly associated with poor clinical outcome in OPSCC patients. In the same patient cohort, IL-6 overexpression was also associated with poor survival. Interestingly, high expression of IL-6 was directly correlated with nuclear PRMT5 localization, thereby suggesting that IL-6 might be promoting PRMT5 nuclear translocation. To examine the effect of IL-6 on PRMT5 nuclear localization, we performed nuclear/cytosolic fractionation and immunofluorescence staining experiments using head and neck cancer cell lines. Our results show that IL-6 treatment of CAL27 cells markedly enhanced nuclear translocation of PRMT5 (Figure 7A–7B). Similar IL-6-mediated nuclear translocation of PRMT5 was observed in another head and neck cell line (UM-SCC-74B; Figure 7C).

## DISCUSSION

The role of PRMT5 in cancer, particularly in regulating the expression of key tumor suppressor genes, has been extensively studied [14, 15, 18]. However, there are conflicting reports about the significance of nuclear versus cytoplasmic localization of PRMT5. In this study, we examined the PRMT5 subcellular expression pattern in surgically treated oropharyngeal squamous cell carcinoma (OPSCC) samples and correlated that with survival, p16 status, and other clinical and pathological variables. Our results show that nuclear PRMT5 expression was highly predictive of poor overall survival in OPSCC patients and it directly correlated with cyclin D1 and IL-6 expression. Nuclear PRMT5 expression has been found to be directly associated with invasive colorectal carcinoma and highly proliferative breast cancer [34, 35]. In contrast, cytoplasmic PRMT5 expression was recently shown to be directly correlated with poor prognosis in lung adenocarcinoma [36, 37]. It is well established that PRMT5 mediates its biological effects by methylating a number of different proteins including p53, myelin basic protein and SM proteins [38–40]. However, it is the histone dimethylation, particularly symmetric dimethylation of histone 4(R3) and histone 3(R8), that is associated with transcriptional repression [16, 18]. Histones are exclusively localized and perform their biological functions in the nucleus of eukaryotic cells. Therefore, PRMT5 translocation to nucleus is essential for histone methylation and subsequent repression of tumor suppressor genes. We had recently shown that IL-6 overexpression promotes tumor metastasis in head and neck cancer by inducing epithelial-mesenchymal transition (EMT) [41]. In this study, we observed that nuclear PRMT5 expression is directly correlated with high IL-6 expression, thereby suggesting that IL-6 might be promoting PRMT5 translocation to nucleus. Indeed, our mechanistic experiments showed that IL-6 treatment of head and neck cancer cells promote PRMT5 translocation to nucleus. PRMT5 subcellular localization is also regulated by binding partners. Recently, IL-6 target protein Snail was shown to form a complex with PRMT5-MEP50. This complex was translocated to the nucleus by interaction with LIM protein Ajuba [17]. Snail recruits this complex to the E-cadherin proximal promoter site, resulting increased H4R3 methylation. These results suggest that transcriptional repression of E-cadherin and EMT by IL-6/snail is dependent on nuclear translocation of PRMT5 and its methyltransferase activity [41]. A number of studies have shown that IL-6 promotes head and neck cancer cell proliferation, migration, survival, invasion, epithelial to mesenchymal transition (EMT), stem cell expansion, and chemoresistance via the activation of JAK/STAT3 signaling pathway [41–43]. Recently, JAK2V617F (a constitutively active JAK2 mutant) was shown to phosphorylates PRMT5 in myeloproliferative neoplasms [44]. It is possible that PRMT5 nuclear translocation is promoted by IL-6/JAK2 mediated PRMT5 phosphorylation.

Over the past decade, it has become apparent that the incidence of “classic” tobacco/alcohol-induced HNSCC has declined, but at the same time, HNSCC caused by HPV has risen sharply [45]. HPV16 is the most prevalent subtype and it accounts for ≈90% of HPV-related HNSCC [46, 47]. Intriguingly, patients with HPV-related HNSCC tend to have far better prognosis than HPV-negative counterparts [9, 10]. A number of potential models have been proposed to explain this clinical outcome disparity between HPV-positive versus HPV-negative patients. However, we still know very little about the precise molecular mechanism(s) that could explain this clinical outcome disparity. In this study, we show an inverse correlation between p16-positivity and nuclear PRMT5 expression. These results suggest that differential nuclear PRMT5 expression might be one of the contributing factors for this prognosis disparity in HPV-positive versus HPV-negative patients. It is possible that high expression of nuclear PRMT5 in HPV-negative tumors is due to the high expression of IL-6 in these tumors as compared to HPV-positive tumors [48].

Cyclin D1 is a key regulator of cell cycle and is often overexpressed in numerous cancer types including breast, colon, lung and head and neck [32, 49]. Our results suggest that high cyclin D1 expression is correlated with poor patient outcome and directly associated with nuclear PRMT5 expression. This could be due to PRMT5-mediated enhanced expression of positive regulators of G1 phase including cyclin D1 [27]. There seems to be a positive feed-back loop between PRMT5 activity and cyclin D1. In addition to PRMT5 increasing cyclin D1 expression, cyclin D1 in turn enhances PRMT5 methyltransferase activity by phosphorylating MEP50 that leads to the formation of MEP50-PRMT5 complex [49]. Taken together, our results suggest that nuclear PRMT5 is a marker for poor clinical outcome in OPSCC. Our mechanistic studies suggest that IL-6 promotes PRMT5 translocation to nucleus. Inhibitors designed to block PRMT5 nuclear translocation and its transcriptional regulatory function may lead to the development of novel therapeutic strategies for the treatment of head and neck cancer.

## MATERIALS AND METHODS

### Study population

Oropharyngeal squamous cell carcinoma (OPSCC) tissue specimens were obtained from surgical resections of patients at The Ohio State University James Cancer Hospital and Solove Research Institute between 2002 to 2009. All patients underwent surgical resection as a first line of therapy with a curative intent. This was followed by no additional treatment or adjuvant chemotherapy and/or radiotherapy as needed. The Ohio State University Institutional Review Board approved a retrospective analysis study of these specimens and a waiver of HIPAA authorization was obtained. Patient characteristics, including age, race, gender, marital status, smoking status, pathological variables including tumor size, nodal status, AJCC stage, extracapsular spread, perineural invasion and clinical variables including survival and recurrence outcomes were recorded.

### Tissue microarray (TMA)

Paraffin-embedded archival tissue blocks and their matching H&E-stained slides were retrieved from the Department of Pathology. A pathologist marked the areas with cancer and adjacent normal on the H&E slides. Representative regions (three cores of tumor tissue and one core of adjacent normal tissue) were sampled using a 0.6-mm punch on a master TMA blocks. The TMA's were constructed by the Histology Core in the Department of Pathology. Unstained sections were cut and used for immunohistochemical staining.

### Immunohistochemistry and scoring

TMA slides were stained to assess the tumor expression of PRMT5, p16, cyclin D1 and IL-6, using immunohistochemistry as previously described [10]. Briefly, slides were deparaffinized in xylene, and rehydrated in decreasing concentrations of ethyl alcohol. Antigen retrieval was performed in a decloaking chamber (Biocare Medical, LLC, Concord, CA, USA) using antigen unmasking buffer (Dako) for 20 minutes at 120°C. After a 20 minutes cool down period at room temperature, sections were incubated with dual endogenous enzyme block (Dako) for 10 minutes at room temperature. Non-specific binding sites were blocked by incubating with PBS /serum from species in which the secondary antibody was raised. Sections were then incubated with primary antibody (IL-6 for overnight; R&D Systems, PRMT5 for 1 hour; Cell Signaling and Cyclin D1 for 1 hour; Thermo Scientific-Lab Vision) at room temperature. After washes, slides were incubated with biotinylated donkey anti-goat (Jackson Immunoresearch; for IL-6) or, biotinylated anti-rabbit (Vectastain Elite Kit; for PRMT5 and Cyclin D1) for 30 minutes at room temperature. Slides were rinsed in wash buffer and incubated with avidin-biotin complex (Vector Laboratories, Burlingame, CA, USA) for 30 minutes. They were then rinsed in wash buffer and incubated with 3,3’-diaminobenzidine (Sigma). The slides were rinsed in water, counterstained with Mayer's hematoxylin, mounted and coverslipped. p16 expression was determined using the CINtec p16 histology kit (MTM/Roche, Heidelberg, Germany). Stained slides were interpreted by a pathologist who was blinded to treatment outcome at the time of review. For p16, cyclin D1 and IL-6, each core was scored for the stain proportion (0–100%) and intensity (1: none, 2: low, 3: moderate, 4: high). A quick score was generated by multiplying the stain proportion scores with stain intensity scores to obtain values between 0–400. For PRMT5 expression, cores were scored for stain intensity and location of PRMT5 (nuclear, cytoplasmic or none). A tumor was considered to be p16 positive when ≥ 50% of the tumor cells displayed strong and diffuse staining pattern.

### Cell lines and reagents

CAL27 cells were obtained from ATCC (Manassas, VA). UM-SCC-74B cell line was obtained from the laboratory of Dr. Thomas E. Carey at the University of Michigan.[50] The identity of both the tumor cell lines was confirmed by STR genotyping (AmpFLSTR Identifiler Kit, Applied Biosystems, Carlsband, CA). The tumor cell lines were cultured in DMEM supplemented with 10% fetal bovine serum containing 1% penicillin/streptomycin (Invitrogen, Carlsbad, CA) and 1% Non-essential amino acids. Recombinant IL-6 was obtained from PeproTech (Rocky Hill, NJ). Primary antibodies against PRMT5 and Lamin/AC were purchased from Cell Signaling (Danvers, MA) and GAPDH was purchased from Millipore (Billerica, MA).

### Cytoplasmic and nuclear protein extraction

Cytoplasmic and nuclear fractions from tumor cells were separated using the NE-PER Nuclear & Cytoplasmic extraction kit (Pierce, Rockford, IL). After IL-6 treatment, cells were harvested and washed with PBS. Cell pellet was treated with cell membrane lysis reagents from the extraction kit to release the cytoplasmic contents. The cytoplasmic proteins were collected by centrifugation leaving the intact nuclei in the pellet. The nuclear pellet was washed with PBS to reduce carryover of the cytoplasmic proteins to the nuclear protein fraction. Nuclear lysis buffer was then added to the pellet to lyse the nuclei and release the nuclear proteins. Reducing agent and loading buffer were then added to the cytoplasmic and nuclear extracts and analyzed by Western blotting.

### Western blot analysis

Whole cell lysates or cytosolic/nuclear protein fractions were separated by 4–12% NuPAGE Bis-Tris gels (Invitrogen, Carlsbad, CA) and transferred onto PVDF membranes using NuPAGE transfer buffer (Invitrogen). To block nonspecific binding, membranes were incubated with 5% Milk in Tris buffered saline containing 0.1% Tween-20 (TBST) or 3% BSA in TBST for 1 hour at room temperature. The blots were then incubated with the respective primary antibody in TBST + 5% Milk or 3% BSA according to manufacturer's instructions at 4°C overnight. After washing with TBST, the blots were incubated with horseradish peroxidase-conjugated sheep anti-mouse IgG (1:3,000) or with donkey anti-rabbit IgG (1:4,000) for 1 hour at room temperature. An ECL-plus Western blotting substrate (Thermo Scientific-Pierce, Rockford, IL) was used to detect specific protein bands.

### Immunofluorescent staining

Tumor cells were cultured in Labtech chambers in serum free medium for 2 hours and then treated with recombinant IL-6 (50 ng/ml) for 30 minutes. At the end of incubation, cells were fixed with 4% paraformaldehyde for 15 minutes at room temperature and permeabilized by treating with 100% methanol for 10 minutes at –20°C. Next, slides were washed with PBS, blocked with normal goat IgG for 1 hour and incubated overnight at 4°C with mouse anti-PRMT5 antibody. After washing with PBS, chamber slides were incubated with secondary antibodies (goat anti-mouse-IgG-Alexa Fluor 488). Chamber slides were then mounted with ProLong Gold antifade reagent with DAPI (Invitrogen). The fluorescent images were captured using Nikon Eclipse 80i microscope with DS-Ri1 camera at 600X magnification and overlaid using NIS-Elements-Basic Research software (Nikon, Melville, NY).

### Statistical analyses

Overall survival was defined as time from the date of surgery to date of death, with patients alive at the date of last observation censored. Cox proportional hazards models were used to assess univariate associations of biomarkers or patient characteristics as predictors for death. Unadjusted hazard ratios (HR) and 95% confidence intervals (CI) are reported. Multivariable models were built to estimate adjusted HRs which let us asses marker effects (nuclear PRMT5, p16, Cyclin D1, and IL-6) beyond the effects of T stage, N stage, AJCC stage, smoking status and gender. To assess dual marker interactions, comparisons of survival curves were evaluated using the log-rank test with adjustments for multiple comparisons made by Bonferonni corrections. Mann-Whitney tests were used to assess associations between biomarkers (p16, Cyclin D1, and IL-6) or demographics (smoking status) and nuclear PRMT5 or Cyclin D1 expression. All analyses were conducted in SAS, version 9.3 (SAS Institute, Cary, North Carolina).
